# Supplementary material for: Exploring the chemical composition, in vitro and in silico study of the anticandidal properties of annonaceae species essential oils from the Amazon
Source: PLoS One. 2023 Aug 24;18(8):e0289991. doi: 10.1371/journal.pone.0289991 (PMC10449155; doi:10.1371/journal.pone.0289991)
Supplement: Attachment — Submitted filename: Review PONE-D-23-15730.pdf [file pone.0289991.s001.pdf]

The manuscript PONE-D-23-15730 entitled "Exploring the Chemical Composition, In Vitro and in Silico Study of the Antimicrobial Properties of Annonaceae Species Essential Oils from the Amazon" reports the composition of the essential oils (EOs) of several plants of the Annonaceae family and explored their potential interaction with the proteins Sterol 14α-Demethylase Cytochrome P450, as their molecular target using a molecular docking approach. The Knowledge added by the manuscript is qualified in the context of the antimicrobial activity of the tested EOs, but just include the molecular docking is not sufficient to improve the value of the study, more validation and exploitation on the use of these EOs is required to considerer this manuscript to be published.

Following the suggestion of minor alterations

1. There is a link in the title to an article (**https://doi.org/10.3390/molecules17089540**)
2. Line 36 MFC first in full
3. Line 36-37 rewrite the sentence is Grammarly confused
4. Line 41 change ntimicrobial to antimicrobial
5. Line 82 change were use to were used
6. Line 92 indicate the first and surname of J. O.
7. Line 110 revise the order of your references here and all over the manuscript
8. Line 133 indicate the volume used and the respective concentration for nystatin.
9. Line 146 eliminate in a bacteriological incubator
10. Line 148 and this concentration was considered the Minimum Inhibitory Concentration?
11. Line 152 MIC in full first
12. Line 153 what dilution? what the authors diluted?
13. Line 155 this is not clear. Rewrite this section. Moreover, the cited reference is in Portuguese.
14. Line 234 check the sentence
15. Line 272 As the authors can observe from the literature cited, is recommended to use the concentration of the EO in ug/mL or mg/mL, and not v/v.
16. Line 272-278 Line 266-278. A proper discussion must be provided, not a statement of the results reported by other studies.
17. Line 285 what paper? the current study or the previous mentioned?
18. Figure 1 and 2 The legend of the Figures must be at the bottom of the Figure.
19. Line 385 the format of the references requires a deep revision.
